# *Mycobacterium tuberculosis*-Induced Polarization of Human Macrophage Orchestrates the Formation and Development of Tuberculous Granulomas *In Vitro*

**DOI:** 10.1371/journal.pone.0129744

**Published:** 2015-06-19

**Authors:** Zikun Huang, Qing Luo, Yang Guo, Jie Chen, Guoliang Xiong, Yiping Peng, Jianqing Ye, Junming Li

**Affiliations:** 1 Department of Clinical Laboratory, the First Affiliated Hospital of Nanchang University, Nanchang, Jiangxi, China; 2 Department of Clinical Laboratory, Jiangxi Chest Hospital, Nanchang, Jiangxi, China; 3 Department of Tuberculosis, Jiangxi Chest Hospital, Nanchang, Jiangxi, China; The Catholic University of the Sacred Heart, ITALY

## Abstract

The tuberculous granuloma is an elaborately organized structure and one of the main histological hallmarks of tuberculosis. Macrophages, which are important immunologic effector and antigen-presenting cells, are the main cell type found in the tuberculous granuloma and have high plasticity. Macrophage polarization during bacterial infection has been elucidated in numerous recent studies; however, macrophage polarization during tuberculous granuloma formation and development has rarely been reported. It remains to be clarified whether differences in the activation status of macrophages affect granuloma formation. In this study, the variation in macrophage polarization during the formation and development of tuberculous granulomas was investigated in both sections of lung tissues from tuberculosis patients and an *in vitro* tuberculous granuloma model. The roles of macrophage polarization in this process were also investigated. *Mycobacterium tuberculosis* (*M*. *tuberculosis*) infection was found to induce monocyte-derived macrophage polarization. In the *in vitro* tuberculous granuloma model, macrophage transformation from M1 to M2 was observed over time following *M*. *tuberculosis* infection. M2 macrophages were found to predominate in both necrotic and non-necrotic granulomas from tuberculosis patients, while both M1 and M2 polarized macrophages were found in the non-granulomatous lung tissues. Furthermore, it was found that M1 macrophages promote granuloma formation and macrophage bactericidal activity *in vitro*, while M2 macrophages inhibit these effects. The findings of this study provide insights into the mechanism by which *M*. *tuberculosis* circumvents the host immune system as well as a theoretical foundation for the development of novel tuberculosis therapies based on reprogramming macrophage polarization.

## Introduction

Tuberculosis, caused by *Mycobacterium tuberculosis*, remains one of the major causes of mortality worldwide and is responsible for 1–2 million deaths each year [1, 2]. In most cases, *M*. *tuberculosis* establishes latent infection in humans. Only 5%–10% of infected individuals develop clinical symptoms, indicating that the host immune system constrains *M*. *tuberculosis* replication in all other infected individuals. On the other hand, *M*. *tuberculosis* produces neither toxins nor evasive enzymes; thus, the pathological lesion of tuberculosis also results from the host’s immune response. Therefore, the immune response of the host critically influences the progression of *M*. *tuberculosis* infection.

The most direct and focused site of interplay between the host immune system and *M*. *tuberculosis* is the tuberculous granuloma, which is one of the main histological hallmarks of tuberculosis. The tuberculous granuloma is an organized structure consisting mainly of macrophages, epithelioid cells, multinucleated giant cells and T lymphocytes [3]. It is generally considered that the main function of the tuberculous granuloma is to localize the infected bacteria and to prevent the spread of infection. It has been reported that the development and prognosis of tuberculous granulomas determine the outcome in *M*. *tuberculosis* infections to some extent [4–6], although the details of this function remain unclear. In most cases, macrophages cannot completely eradicate *M*. *tuberculosis*, but endocytose the bacilli to restrain their replication. More importantly, following the phagocytosis of *M*. *tuberculosis*, macrophages secrete a variety of chemoattractant cytokines, which help recruit uninfected macrophages, monocytes and lymphocytes from the circulation to the site of infection, initiating the formation of tuberculous granulomas [7]. Therefore, deciphering the functions of the various types of macrophages in tuberculous granulomas is important to elucidate the mechanism of tuberculous granuloma formation and development, and to improve our understanding of the general host immune response against *M*. *tuberculosis*.

Macrophages are a complex cell population with a high level of plasticity [8]. Once activated, macrophages can present a myriad of different, sometimes contradictory, functions such as promotion of inflammatory responses and angiogenesis, tumor growth inhibition, tissue remodeling and cell destruction [9–11]. Previous studies have demonstrated that the function of macrophages correlates with their microenvironment, especially the types and concentrations of cytokines. Following the Th1/Th2 classification system, the terms M1 and M2 were introduced into macrophage nomenclature to define the two main polarized activation states of macrophages.

Generally, M1 polarization of macrophages is induced by LPS or IFN-γ (also known as classical activation), leading to the proinflammatory response and increased microbicidal and tumoricidal capacity. In microenvironments dominated by IL-4 or IL-13, macrophages undergo M2 polarization (also known as alternative activation), which mediates tissue repair and immune escape of pathogens and tumors, leading to persistent infection and tumor development [9,12]. In addition to their different functionalities, M1 and M2 macrophages display different phenotypes and cytokines secretion profiles. In humans, M1 macrophages are characterized by high levels of CXCL10, CXCL11, CCR7, TNF-α, IL-6 and IL-12, while M2 macrophages exhibit elevated levels of CCL17, CCL18 and IL-10 [10].

Recent studies have shown that the activation status or polarization of macrophages is critical for the development of host immune responses against invading pathogens [13]. However, some pathogens, such as hepatitis B virus [14], facilitate their immune escape and pathogenesis by modulating macrophage polarization. Recently, it was reported that *M*. *tuberculosis* also has the potential to modulate macrophage polarization. In macaque and human tuberculous granulomas, Mattila et al. [15] found substantial coexpression of proinflammatory and anti-inflammatory macrophage markers in granulomatous macrophages. This is the first report focusing on the polarization of granulomatous macrophages in a *M*. *tuberculosis* infection model to demonstrate that macrophage activation in tuberculous granuloma is not binary, but occurs along a spectrum. However, due to the restriction in macaque and human specimens, Mattila et al. focused mainly on the observation of phenotypic markers of macrophages in tuberculous granulomas at a fixed time-point. The variation in the functional markers and polarization status of granulomatous macrophages at different stages of infection, and the impact on the formation and development of tuberculous granulomas are still unclear. Most recently, Marino et al. [16] predicted that macrophage polarization drives granuloma outcome during *M*. *tuberculosis* infection using a computational biology approach, although this hypothesis requires confirmation.

In this study, the variation in macrophage polarization status during the formation and development of tuberculous granulomas was investigated in both histological analyses of sections of lung tissues from tuberculosis patients and the *in vitro* tuberculous granuloma model. A preliminary investigation of the role of macrophage polarization in the formation and development of tuberculous granulomas was also conducted. To our knowledge, this is the first report describing the dynamic changes in macrophage polarization status within tuberculous granuloma. Additionally, our study revealed that the polarization of macrophages orchestrates the formation and development of the tuberculous granuloma, which might improve our understanding of the role of macrophages role in immune responses against tuberculosis.

## Materials and Methods

### Human samples and cell culture

Eighteen lung tissue resection samples were collected from active tuberculosis patients. Each tissue sample was divided into three parts; one part was paraffin-embedded and ultrathin sections were prepared for histopathological and acid-fast staining examination; another part was frozen and sliced with a cryostat for immunohistochemical analysis; the last part was divided into the granulomatous tissue and the surrounding non-granulomatous tissue under a dissecting microscope. Total RNA was extracted from these two divided tissues using TRIzol reagent (Invitrogen, USA) for quantitative PCR analysis.

Peripheral blood samples were collected from healthy PPD-negative donors with no previous contact with tuberculosis patients. Peripheral blood mononuclear cells (PBMCs) were isolated from these blood samples according to previously reported procedures [17,18]. Briefly, 15 ml whole blood was added to 15 ml of Hank’s balanced salt solution (HBSS; Invitrogen, USA), and then gently layered onto 15 ml Ficoll-Hypaque solution (Sigma, USA) in a 50-ml polypropylene tube. This was then centrifuged at 2,000 rpm for 20 min at 20°C. The cells at the interface were carefully transferred with a Pasteur pipette to a fresh 50-ml tube and HBSS was added. After further centrifugation at 1,500 rpm for 10 min at 20°C, the supernatant was discarded and cells were resuspended in HBSS. After centrifugation the cells were then suspended in RPMI 1640 medium (Gibco, USA) at 5×10^6^/ml and cultured at 37°C in a humidified incubator under 5% CO_2_.

To develop monocyte-derived macrophages (MDM), monocytes were purified using anti-CD14 magnetic beads (Miltenyi Biotec, USA) and then cultured in RPMI 1640 medium containing 10% human serum and 0.05% glutamine. MDMs were identified on the basis of morphology and flow cytometric (FCM) evaluation of anti-CD68 staining. CD68-positive cells with typical macrophage morphology were used as MDM in subsequent experiments.

To induce MDM polarization, CD68-positive cells were treated with 10 ng/ml IFN-γ (R&D Systems, USA) and 100 ng/ml of LPS (PeproTech, USA) to achieve M1 polarization, or with 10 ng/ml IL-4 (PeproTech, USA) to achieve M2 macrophage. The non-polarized MDM were cultured without treatment (M0 phenotype). After 48 h, the supernatant was collected and adherent cells were harvested for further analysis.

### Bacterial strains and culture conditions

*M*. *tuberculosis*-GFP was constructed by transforming plasmid pUV15 (a kind gift from Professor Michael Niederweis, Department of Microbiology, University of Alabama, Birmingham, AL, USA) into *M*. *tuberculosis* H37Rv (ATCC 27294) and screening on Middlebrook 7H10 agar (BD company, USA) containing 50 μg/ml hygromycin B.

*M*. *tuberculosis* H37Rv (ATCC 27294) and *M*. *tuberculosis*-GFP were grown to early mid-log phase in Middlebrook 7H9 broth (BD, USA) with or without hygromycin B, supplemented with 10% albumin-dextrose-catalase at 37°C. The bacterial aggregates were disrupted by gentle agitation with 3-mm-diameter glass beads (Merck) and diluted in phosphate-buffered saline (PBS) and allowed to stand for 15 min, before the supernatant were collected and adjusted to an OD_600_ of 0.5 (approximately 10^7^ individualized bacteria/ml). Bacteria were plated for determination of viable counts on Middlebrook 7H10 agar with or without hygromycin B.

### Generation of the *in vitro* model of tuberculous granuloma

The *in vitro* model of tuberculous granuloma was generated as previously reported [17–20]. Briefly, PBMCs isolated according to the method described previously, were placed in a 24-well plate (10^6^ cells/well) (Corning Life Sciences, USA), and cultured in RPMI 1640 medium at 37°C under 5% CO_2_. The cells were then infected with *M*. *tuberculosis* at a multiplicity of infection (MOI) of 1:100 (bacteria/cells). The morphology and size of cellular aggregates were observed and recorded during granuloma formation. To measure the distribution of *M*. *tuberculosis* in this *in vitro* model of tuberculous granuloma, PBMC were also infected with *M*. *tuberculosis*-GFP, stained with rhodamine-phalloidin and analyzed by laser scanning confocal microscopy.

For histological assays, the tuberculous granulomas were harvested following reported procedures [18]. Briefly, after the medium was removed from the *M*. *tuberculosis*-infected cells prepared as described previously, 1 ml 10% formalin fixative was added to each well. After 2 h, cell aggregates were then stained with 100 μl hematoxylin for 2 min. The stain was then removed and 0.5 ml 2% agarose was added into each well. Plates were cooled at 4°C for 10–15 min to allow the agarose to solidify before agarose plugs containing the cellular aggregates were carefully removed from the plates, embedded in paraplast paraffin and sectioned (5 μm thickness) using a microtome

For FCM or RT-PCR analyses, cells were digested with trypsin, collected by centrifugation, and digested with 0.5 mg/ml collagenase (Sigma, USA) and 30 μg/ml DNase I (Sigma). The individualized MDM cells were then purified using the adherence method.

### Histological processing

For hematoxylin and eosin (H&E) staining, the tissue sections were stained with hematoxylin for 10 min. After gentle rinsing in running water for 1 min, the tissue sections were differentiated in 1% hydrochloric acid alcohol solution (1–3 s) followed by staining with eosin (5–10 s). The sections were dehydrated and then soaked in xylene twice before being analyzed under a microscope.

For the acid-fast analysis, the tissue sections were stained with carbol fuchsin at 60°C for 1 h and decolorized with 5% hydrochloric alcohol, prior to staining with methylene blue.

For immunohistochemical analysis, the tissue sections were immersed in 3% H_2_O_2_ and then rinsed with PBS to deactivate the endogenous peroxidase. Non-specific staining was blocked by incubation with 5% bovine serum albumin (Invitrogen, USA) for 30 min. The sections were then incubated with mouse anti-human CD68 monoclonal antibody (ZSGB-Bio, Beijing, China), rabbit anti-human iNOS polyclonal antibody (Abcam, UK) or mouse anti-human CD206 monoclonal antibody (BioLegend, USA) at 4°C overnight. After extensive washing in PBS, the tissue sections were incubated with goat anti-rabbit or goat anti-mouse secondary antibodies (IgG/HRP) at 37°C in darkness for 15 min. After further extensive washing in PBS, the sections were covered with 200 μl DAB (ZSGB-Bio) and incubated for 3–5 min. The sections were then gently rinsed in running water to remove excess DAB, and were stained with hematoxylin for 3 min, before they were differentiated in the 1% hydrochloric acid alcohol solution for 3–5 s and incubated in saturated lithium carbonate solution for 3–5 s. Subsequently, the sections were dehydrated in a gradient alcohol series and soaked in the xylene twice before they were analyzed under a microscope.

### RNA isolation and real-time RT-PCR

Total RNA was extracted using TRIzol reagent (Invitrogen, USA). The cDNA was synthesized with PrimeScript reverse transcription reagents (TaKaRa, Japan). The reverse transcription was carried out at 37°C for 30 min before the reaction was terminated at 85°C for 5 s.

Real-time PCR was carried out in the StepOnePlus thermal cycler (Applied Biosystems) using the SYBR Premix Ex Taq II reagents (TaKaRa) under the following conditions: denaturing for 30 s at 95°C followed by 40 cycles of 15 s at 95°C and 30 s at 60°C and dissociation for 15 s at 95°C followed by 1 min at 60°C and 15 s at 95°C. The primers for targeted genes are listed in **Table 1**. GAPDH was set as the internal control. Each experiment was carried out in triplicate, and the results from each trial were normalized before the analysis of the changes of relative gene expression level was performed.

**Table 1:** Primers for target genes.

Genes	Forward (5′–3′)	Reverse (5′–3′)
*GAPDH*	GCACCGTCAAGGCTGAGAAC	TGGTGAAGACGCCAGTGGA
*CXCL10*	GGCCATCAAGAATTTACTGAAAGCA	TCTGTGTGGTCCATCCTTGGAA
*CXCL11*	CCTTGGCTGTGATATTGTGTGCTA	CCTATGCAAAGACAGCGTCCTC
*CCL17*	TGAGGACTGCTCCAGGGATG	AACGGTGGAGGTCCCAGGTA
*CCL18*	AAGAGCTCTGCTGCCTCGTCTA	CCCTCAGGCATTCAGCTTCA
*CCR7*	GGTGGTGGCTCTCCTTGTCATT	GCTTTAAAGTTCCGCACGTCCTT

### Flow cytometry analysis and ELISA

Cells were stained for cell surface markers by incubation for 30 min at room temperature with antibodies against CD86 (PE-CD86, clone IT2.2), HLA-DR (FITC-HLA-DR, clone L243), CD206 (PE-CD206, clone 19.2) and CD163 (FITC-CD163, clone GHI/61). Each antibody was titered to achieve optimal cell staining. Appropriate isotype control antibodies (PE-IgG2b and FITC-IgG2b) were used at the same concentration. All antibodies were purchased from eBioscience Company (USA). After staining, cells were washed twice with PBS and resuspended in 0.5 ml PBS before the FCM analysis (Beckman Coulter, USA). Each experiment was repeated at least three times, and all data were analyzed with FlowJo Software (Tree Star). Cells were gated on the macrophage population based on size (forward-angle light scatter) and density (side-angle light scatter).

### Cytokine assays

The supernatant from cell culture plates was collected at several time-points. The collected supernatants were filtered with 0.22 μm low protein-binding filter and then stored at -80°C. Cytokines IL-6, IL-10, IL-12 and TNF-α in the supernatants were measured using ELISA kits (R&D Systems) according to the manufacturer’s instructions.

### Examination of bactericidal activity

Cells were infected with *M*. *tuberculosis* (MOI = 5) and cultured at 37°C under 5% CO_2_ for 4 h. After centrifugation, the supernatant was collected and the infected cells were washed three times with pre-warmed HBSS to remove the non-phagocytized bacteria. The cells were lysed with 0.05% SDS in PBS at several time-points. Bacteria in the supernatants and cell lysates were collected by centrifugation. The collected bacteria were mixed and used to inoculate Middlebrook 7H10 solid medium containing 10% OADC (BD). The inoculated bacteria were cultured at 37°C for 3 weeks before colony counting.

### Ethics statement

Written informed consent was obtained from all participants before their enrollment into the study. The experiments conducted in this study were approved by the Ethics Committee of the First Affiliated Hospital of Nanchang University (China). The Institutional Ethics Committee specifically approved this study (Approval number: 2011–003). All investigation protocols were carried out according to the principles of the Declaration of Helsinki.

### Statistical analysis

All data were analyzed with SPSS17.0 software, and results were recorded as mean ± standard deviation (SD). Comparisons between two groups were performed by two-tailed unpaired Student’s *t*-test and all graphs were generated using Graph Prism 5.0 software (San Diego, CA, USA). *P* < 0.05 was considered to indicate statistical significance.

## Results

### *M*. *tuberculosis* induces polarization of human monocyte-derived macrophages *in vitro*

**Fig 1:**
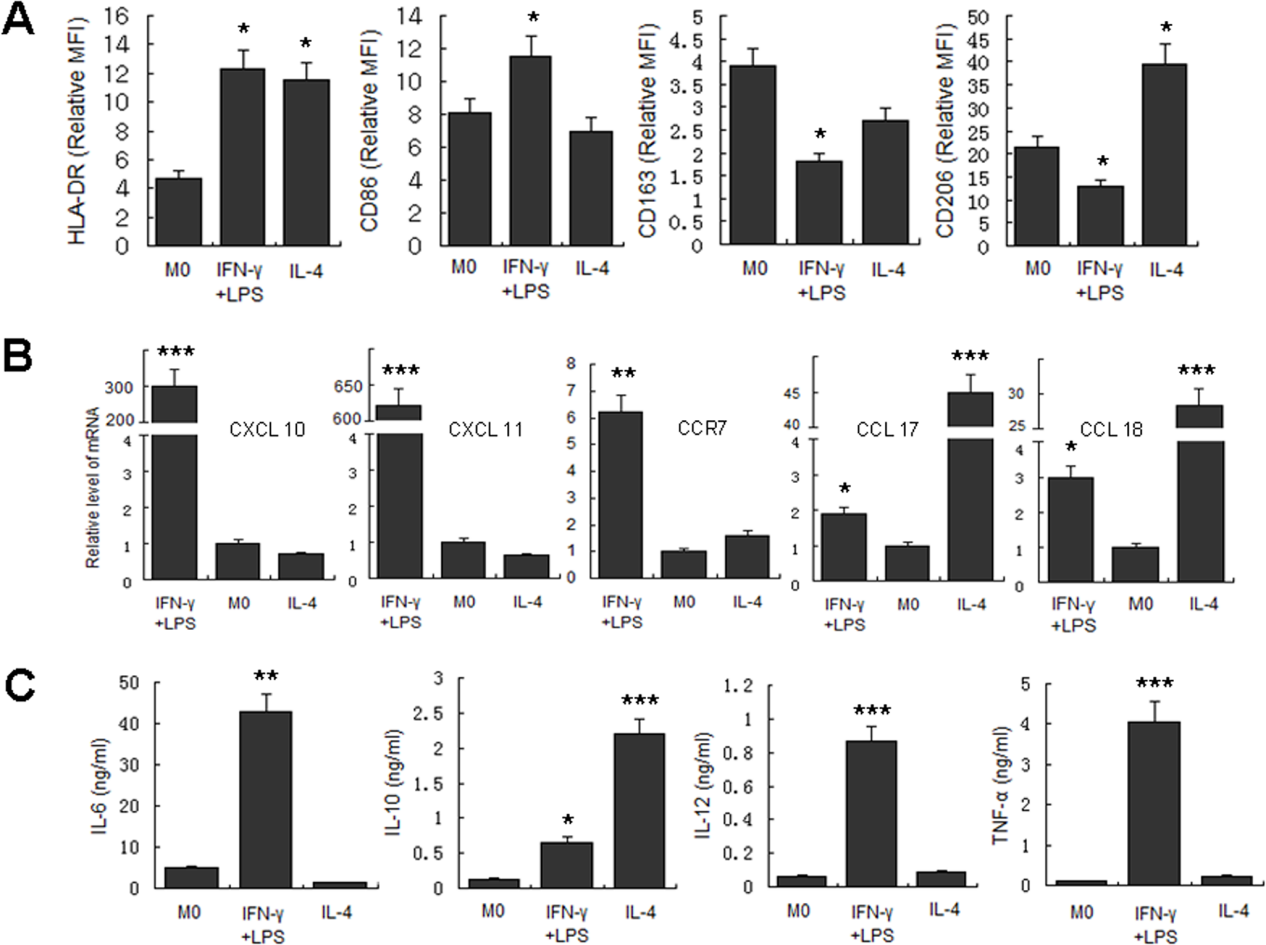
Determination of polarization markers of differently activated MDM. MDM were activated by IFN-γ/LPS, IL-4 or left untreated for 2 days. Markers reported for M1 and M2 macrophages were determined. A. Phenotypic markers were measured by FCM. Mean fluorescent intensity (MFI) was measured and depicted as relative expression as compared to isotype control. Represented is the mean of three donors ±SD. Significance was determined as compared to untreated group. *P < 0.05, **P < 0.01, ***P < 0.001. B. Effector molecules of differently activated macrophages were determined by real-time RT-PCR. Represented is mean ±SD of relevant level of mRNA in activated MDM compared to M0 macrophage. C. Cytokines in the supernatant were measured by ELISA. Represented is the mean of three donors ±SD. Significance was determined as compared to untreated group. *P < 0.05, **P < 0.01, ***P < 0.001.

Before the exploration of macrophage polarization during the course of *M*.*tuberculosis* infection, we first determined the phenotypic and functional markers associated with human M1 and M2 macrophages, including CD86, CD163, CD206, HLA-DR, CXCL10, CXCL11, CCR7, CCL17, and CCL18, as well as the expression of related cytokines in the culture supernatant, including IL-6, IL-10, IL-12, and TNF-α. The results showed that following IFN-γ/LPS induction, the expression of HLA-DR, CD86, CXCL10, CXCL11, and CCR7 significantly increased, and the levels of IL-6, IL-12, and TNF-α in the culture supernatant were also significantly enhanced. On the other hand, IL-4 induction of MDM upregulated the expression levels of molecular markers such as HLA-DR, CD206, CCR7, CCL17, and CCL18. Expression of IL-10 in the culture supernatants was also significantly increased (Fig 1). Based on these findings, CD86, CXCL11, and TNF-α were selected as M1 molecular markers in subsequent studies, and CD206, CCL17, and IL-10 were used as molecular markers for M2.

**Fig 2:**
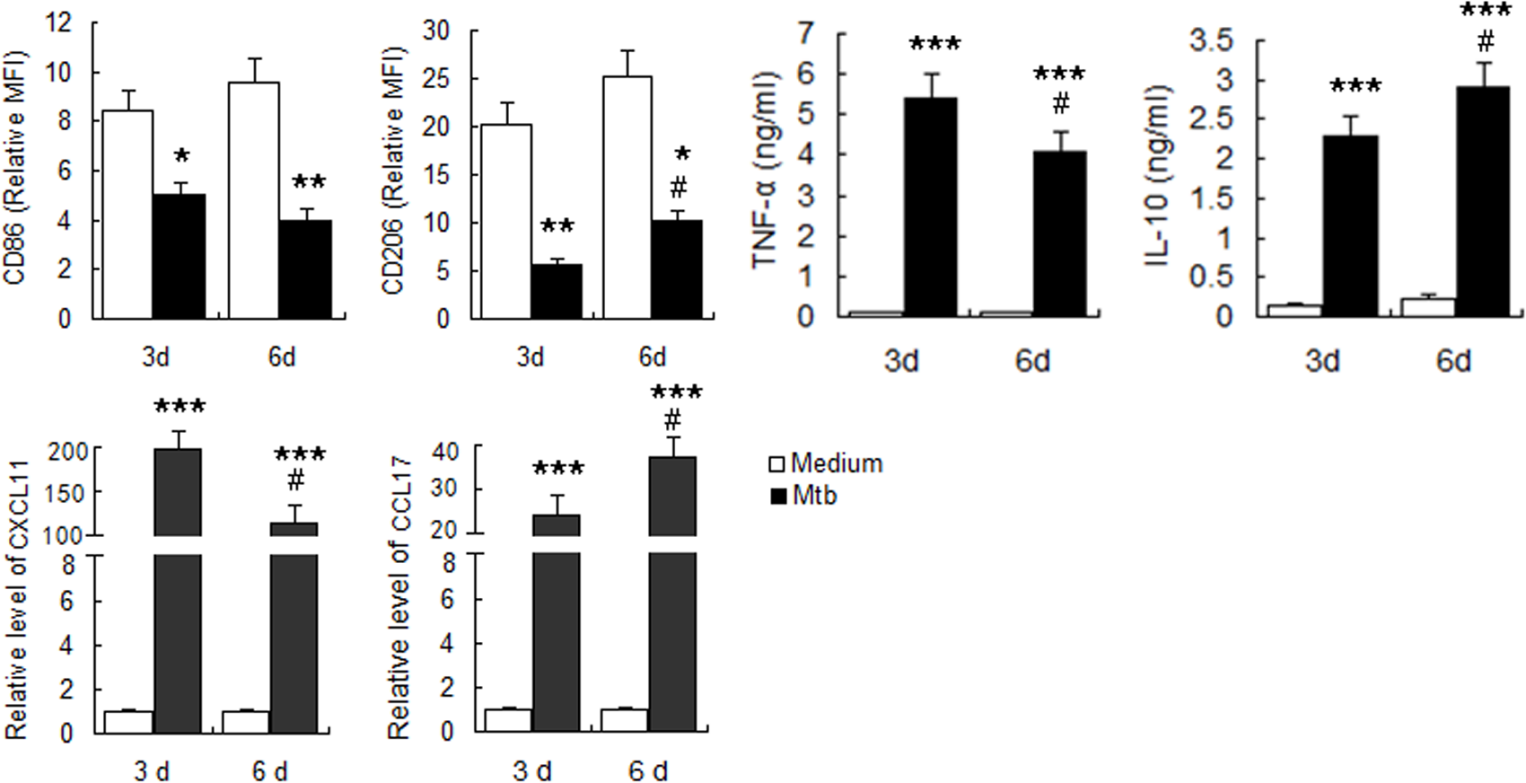
Determination of polarization markers of *M*.*tuberculosis* infected MDM. MDM were infected with *M*.*tuberculosis* H37Rv at MOI of 5 and polarization markers were determined at indicated times. A. Phenotypic markers were measured by FCM. MFI was measured and depicted as relative expression as compared to isotype control. Represented is the mean of three donors ±SD. Significance was determined as compared to uninfected group. *P < 0.05, **P < 0.01, ***P < 0.001. B. Effector molecules of macrophages were determined by real-time RT-PCR. Represented is mean ±SD of fold change of mRNA level in infected macrophages to uninfected MDM. C. Cytokines in the supernatant were measured by ELISA. Represented is the mean of three donors ±SD. Significance was determined as compared to uninfected group. *P < 0.05, **P < 0.01, ***P < 0.001.Significance in corresponding marker expression between 6d and 3d was also calculated and represented by the following symbols: ^#^P < 0.05.

Next, we investigated the activation state of human MDMs and changes at different stages of infection with *M*. *tuberculosis* H37Rv strain. The results showed that the expression of CD86 and CD206 was significantly reduced at three days post-infection compared with that in the uninfected M0 cells. Expression of other markers, including CXCL11, CCL17, TNF-α and IL-10, were significantly upregulated, indicating a mixed activation. With increased time of infection, the expression of M1 macrophage-related markers, including CD86, CXCL11, and TNF-α decreased, while the expression of M2 macrophage-related markers including CD206, CCL17 and IL-10 gradually increased (Fig 2). These results suggested that *in vitro* infection of human MDMs with *M*. *tuberculosis* induces mixed M1/M2 activation, with a gradual transformation to M2 with increased time of infection.

### Macrophages undergo M1 to M2 transformation in the *in vitro* model of tuberculous granuloma

**Fig 3:**
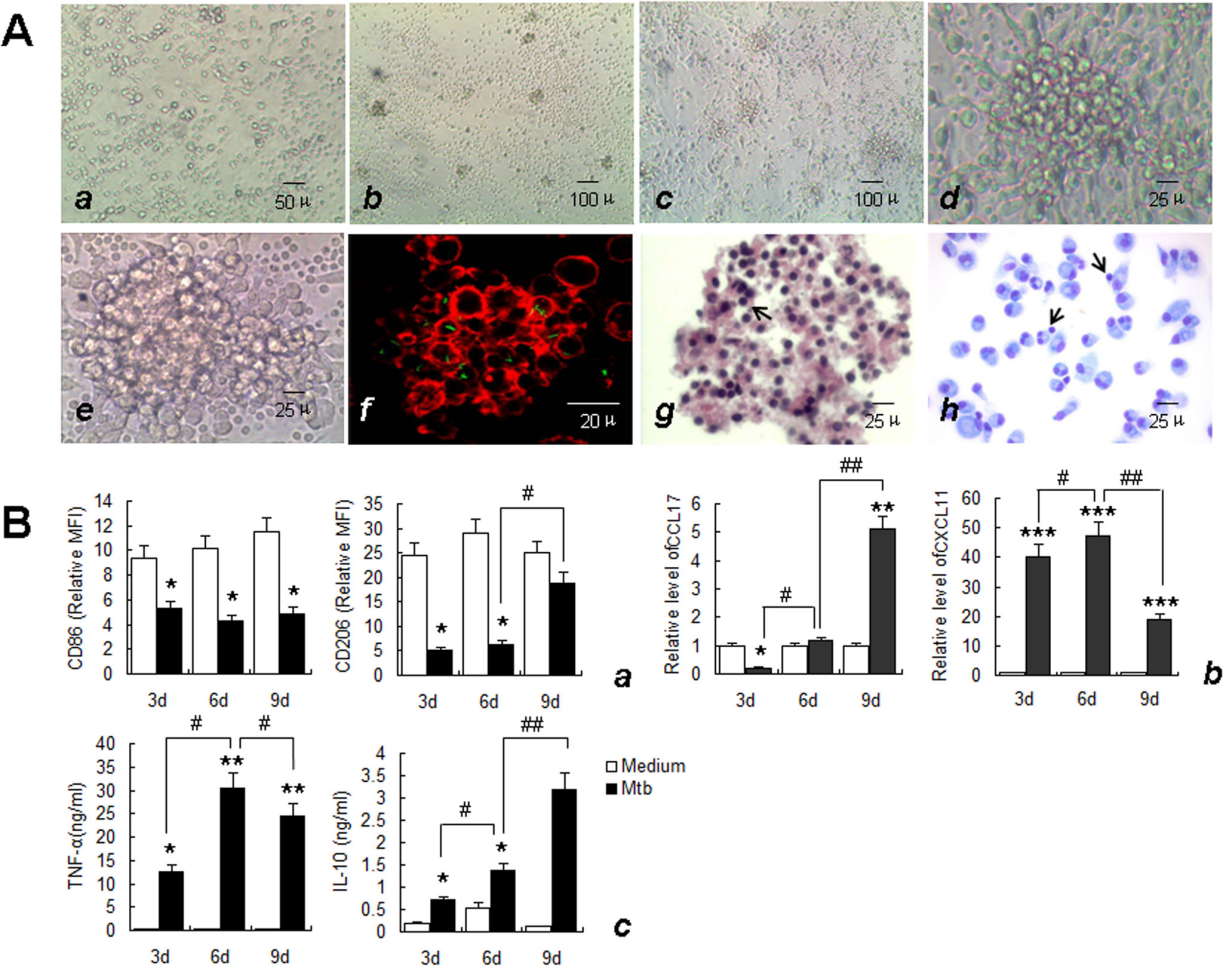
Characteristics of *in vitro* tuberculous granuloma model and the expression of granulomatous macrophage markers. A. Uninfected PBMCs at day 6 (a, ×200) and the morphology and structure of in vitro tuberculous granuloma model at day 3 (b, ×100), day 6 (c, ×100; d, ×400) and day 9 (e, ×400) in light microscopy. The actin filament in cells was labeled with rhodamine-phalloidin and the bacteria between and possibly within the cells was shown in green (f), (×1000). There were mainly macrophages and some lymphocytes (arrows) in granuloma observed by HE (g), (×400), and May-Grünwald-Giemsa staining (h), (×400). Size scale is indicated. B. Supernatant was collected and Macrophages were isolated from in vitro tuberculous granuloma as described in Materials and Methods at indicated times. The phenotypic markers on macrophages were determined by FCM. MFI was measured and depicted as relative expression as compared to isotype control. Represented is the mean of three donors ±SD. (a). Effector molecules were determined by real-time RT-PCR. Represented is mean ±SD of relative level of mRNA in activated MDM compared to M0 macrophage (b). Cytokines in the supernatant were measured by ELISA. Represented is the mean of three donors ±SD (c). Significance was determined as compared to untreated group. *P < 0.05, **P < 0.01, ***P < 0.001. Significance in corresponding marker expression between 9d, 6d and 3d was also calculated and represented by the following symbols: ^#^P < 0.05, ^# #^P < 0.01.

The activation status of macrophages during the formation and development of granulomas was investigated using the *in vitro* model of tuberculous granuloma established by *M*. *tuberculosis* H37Rv infection of human PBMC according to methods described previously [17–20]. The formation and development of granulomas was monitored by light microscopy to day 9 post-infection. Visible cell aggregation occurred on day 3 post-infection with *M*. *tuberculosis* (Fig 3A, *b*). With prolonged infection, the volume of cell aggregates gradually increased, and granuloma-like structures with multilayer cell aggregations (Fig 3A, *c-e*) were observed on day 6. To better observe the distribution of *M*. *tuberculosis* in this *in vitro* model, PBMCs infected with *M*. *tuberculosis*-GFP and stained with rhodamine-phalloidin were observed using laser scanning confocal microscopy. GFP-labeled *M*. *tuberculosis* was found to be distributed between, and possibly within, the aggregated cells (Fig 3A, *f*). No cellular aggregation was observed in the control groups at this stage. To analyze the cellular components of this *in vitro* granuloma, the cell aggregates were harvested, sectioned and stained with H&E and May-Grünwald-Giemsa (MGG) staining. Macrophages and lymphocytes were the main cellular components of these structures **(** Fig 3A, *g* and *h* **)**. The histological characteristics and bacterial distribution of these aggregates of cells were consistent with previous reports of *in vitro* tuberculous granulomas and mimic the “true granuloma” [17–20].

The activation status of macrophages in this model was then evaluated. In the early stages of granuloma-like structure formation (on day 3), the expression of the M1 macrophage-associated molecular markers, CXCL11 and TNF-α were significantly upregulated, with the exception of CD86, which was expressed at lower levels, while the expression levels of the M2-related molecular markers CD206 and CCL17 were significantly decreased. IL-10 expression significantly increased compared with that of M0 cells, but was similar to the level in cells following IFN-γ/LPS induction (Fig 3B). Thus, M1 macrophages were predominant in the early stages of granuloma-like structure formation. During the typical granuloma-like structure formation stage (6d), the expression of both M1 and M2-related markers, including CXCL11, CCL17, TNF-α and IL-10 increased significantly compared with that observed in the early (3d) stages, with the exception of CD86 and CD206, which showed no significant changes. With increased time of infection, the expression of M1-type molecular markers such as CXCL11 and TNF-α significantly decreased, with the exception of CD86, which showed no significant change. In contrast, expression levels of the M2-type molecular markers CD206, CCL17 and IL-10 continued to increase (Fig 3B), suggesting that M2 macrophages predominate during the late stages of granuloma-like structure formation.

### Macrophages in granulomatous and non-granulomatous human tissues exhibit differences in polarization status

**Fig 4:**
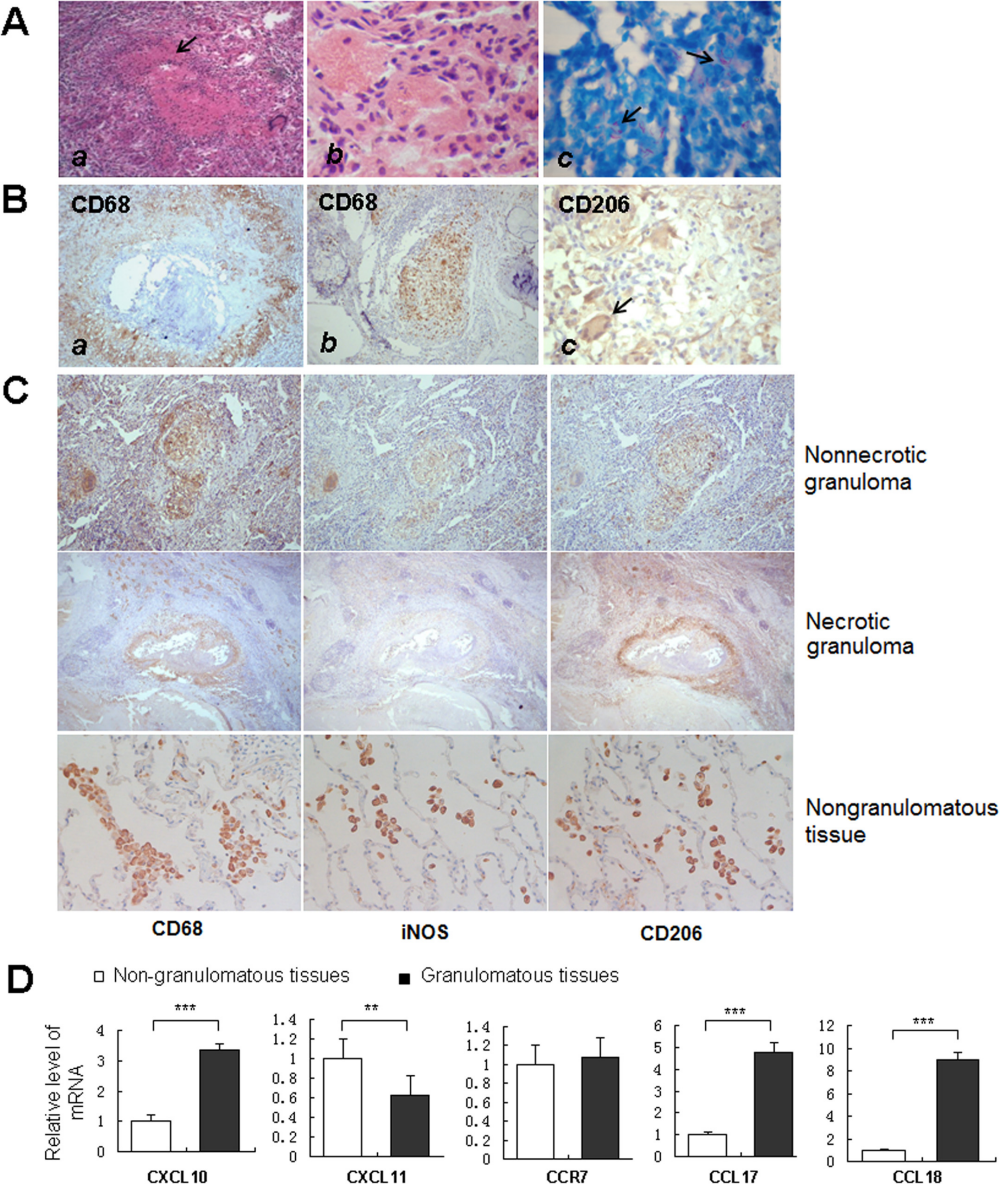
Activation status of macrophages in human tuberculous granuloma. **A.** HE and acid-fast staining of tuberculous granuloma from the human lung tissue of tuberculosis (TB) patients. The structure of tuberculous granuloma with caseous necrosis was obviously observed by HE staining (***a***), (×200). Macrophages with irregular shapes were frequently observed (***b***), (×1000). Bacteria (stained in red, arrows) between and possibly within the granuloma cells were shown by the acid-fast staining (***c***), (×1000). **B.** The immunostaining of aggregate of CD68-positive macrophages in granuloma with caseous necrosis (***a***) or without caseous necrosis (***b***), (×100). CD206-positive multinucleated giant cells were frequently observed (c), (×400). **C.** The immunostaining of serial sections of the same tissue zone showed the expression levels of CD68, CD206 and iNOS in granulomas (×100) and nongranulomatous tissue (×400). D. The levels of CXCL10, CXCL11, CCR7, CCL17 and CCL18 mRNA in granulomatous lung tissues of tuberculosis patients were determined by real-time RT-PCR, in comparison with lung tissues containing no granuloma. The bars represent means ± SD of fold changes of mRNA expression levels in granulomatous tissues (G, showed as black boxes) compared to tissues containing no granuloma (NG, showed as blank boxes). (**P <0.01, ***P <0.001).

Our results indicated that the activation state of macrophages underwent M1-to-M2 transition during the formation and development of *in vitro* tuberculous granulomas. Compared with the *in vitro* model, *in vivo* tuberculous granulomas are formed in a much more complex environment. To further investigate the activation status of macrophages in the tuberculous granuloma, lung tissues from 18 pulmonary tuberculosis patients who received pneumonectomy were collected, sectioned and analyzed by H&E staining, acid-fast staining and immunohistochemistry. Typical tuberculous granulomas were observed in most biopsy tissues as shown by H&E staining (Fig 4A, *a*). Acid-fast staining showed numerous acid-fast *M*. *tuberculosis* (stained red) distributed between, and possibly within, the aggregated cells (Fig 4A, *b*). Immunohistochemical staining for CD68 showed a large number of CD68-positive macrophages in the non-necrotic granulomas and the surrounding regions of caseous necrosis (Fig 4B).

To determine the polarization status of macrophages in the granulomatous lung tissues, as defined by the expression level of inducible nitric oxide (iNOS) and CD206, three serial sections of the same tissue zone were stained with antibodies against CD68, iNOS and CD206. Results showed that macrophages in both necrotic and non-necrotic granulomas were iNOS^med^ CD206^high^, indicating M2 dominant activation. However, macrophages in non-granulomatous tissues showed a different polarization status, characterized by a iNOS^hign^ CD206^high^ phenotype (Fig 4C). The expression levels of CXCL10, CXCL11, CCR7, CCL17 and CCL18 in the tuberculous granuloma were measured by quantitative real-time RT-PCR and were compared with the levels detected in lung tissues without granulomas. The expression levels of M2-related markers (CCL17 and CCL18) significantly increased in the granulomatous lung tissues, whereas the expression of M1-related markers was not consistent between the two groups (Fig 4D). These results demonstrated the coexistence of M1 and M2 macrophages in infected lung tissues, whereas M2 macrophages predominated in granulomatous tissues from patients with active tuberculosis.

### Macrophage polarization modulates granuloma formation and the bactericidal activity of macrophages

**Fig 5:**
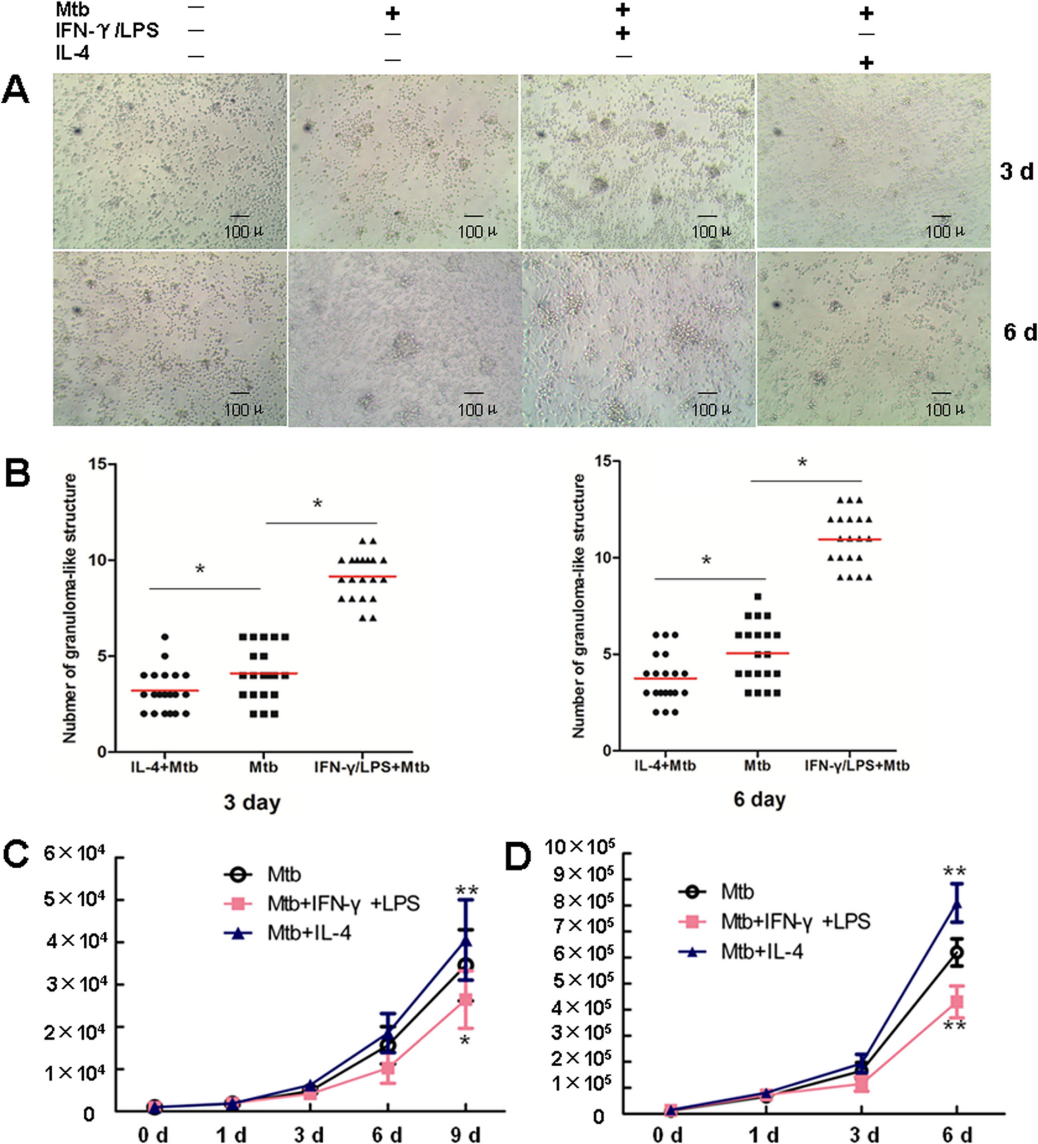
Macrophage polarization modulates granuloma formation and the bactericidal activity of macrophages. **A.** PBMC were left untreated, or infected with *M*.*tuberculosis*, or pretreated with IFN-γ+LPS following infection with *M*.*tuberculosis*, or pretreated with IL-4 following infection with *M*.*tuberculosis* (×100). The aggregate of cells was observed at indicated times in light microscopy. Size scale is indicated. **B**. The number of granuloma-like structures in each group was counted in 20 randomly selected low-power field (LPF, ×100). *P < 0.05. **C**. The *in vitro* model of tuberculous granuloma was generated as described in Materials and Methods. At 48h before *M*.*tuberculosis* infection, PBMC were pretreated with IFN-γ+LPS, or IL-4, or left untreated. Supernatant were collected and cells were lysed at indicated time points. Bacteria in supernatant and cell lysate were collected, mixed and inoculated onto the Middlebrook 7H10 solid medium containing 10% OADC. The bacterial load was determined by CFU counting. Represented is the mean of at least three tests±SD. Significance was determined as compared to untreated group. *P < 0.05, **P < 0.01. **D**. MDM, pretreated with IFN-γ+LPS, or IL-4, or left untreated for 2 day, were infected with *M*.*tb* and cultured as described in Materials and Methods. Supernatant were collected and cells were lysed at indicated time points. Bacteria in supernatant and cell lysate were collected, mixed and inoculated onto the Middlebrook 7H10 solid medium containing 10% OADC. The bacterial load was determined by CFU counting. Represented is the mean of at least three tests±SD. Significance was determined as compared to untreated group. *P < 0.05, **P < 0.01.

To further explore whether the polarization status of macrophage affects the progression of granulomas, IFN-γ+LPS and IL-4 were used to induce M1 and M2 macrophage polarization, respectively, prior to *M*. *tuberculosis* infection. Aggregates of cells were observed by light microscopy at the indicated time-points. Enumeration of the granuloma-like structures in 20 randomly selected low-power fields (LPF, ×100) showed that visible cell aggregation in both groups had at day 3 post-infection. Compared with those in the untreated control group, the quantity of granuloma-like structures in the IFN-γ+LPS-treated group was significantly greater (9.15 per LPF vs. 4.1 per LPF at day 3; 10.95 per LPS vs. 5.05 per LPF at day 6). In contrast, cell aggregation was significantly suppressed in the IL-4-treated group, and significantly fewer granuloma-like structures were observed compared with the untreated control group (3.20 per LPF vs. 4.1 per LPF at day 3; 3.75 per LPS vs. 5.05 per LPF at day 6). With the extension of the infection time, the surface area of the granuloma-like structures in both groups increased. Compared with the non-pretreated infection group, the surface area of the granuloma-like structures was relative larger and the structure tighter in the IFN-γ+LPS-treated group, while the surface area of the granuloma-like structure was relatively smaller and the structure was looser in the IL-4-treated group (Fig 5A). These results indicated that M1 macrophages promote the formation of tuberculous granuloma, while M2 macrophages inhibit this process.

Next, we examined the bactericidal effect of granulomatous cell populations on *M*. *tuberculosis*. IFN-γ+LPS-pretreatment effectively improved the bactericidal function of granulomatous cells on *M*. *tuberculosis*, while IL-4 pretreatment significantly inhibited this bactericidal effect (Fig 5B). To further confirm that the difference in bactericidal activity is related to the polarization state of the macrophages, we used IFN-γ+LPS and IL-4 to induce polarization of human MDM to the M1 and M2 phenotypes, respectively, prior to *M*. *tuberculosis* infection. Measurement of the bacterial load at different time-points post-infection showed significantly lower colony-forming units (CFU) in the M1 (IFN-γ+LPS) group, while that in the M2 (IL-4) group was notably higher (Fig 5C) compared with that in the M0 (un-induced) group. These results were consistent with those obtained using the *in vitro* granuloma model, indicating that M1 macrophages possess higher bactericidal activity, compared with that of M2 macrophages, which is significantly attenuated.

## Discussion

Increasing evidence has shown that macrophages play a dual role in *M*. *tuberculosis* infection. On one hand, macrophages are the main immune effector cells and antigen-presenting cells responsible for anti-tuberculosis immunity; on the other hand, macrophages are a target of *M*. *tuberculosis* and represent a “safe harbor” for the bacteria in infected humans [21,22]. Recent studies suggest that macrophages have great plasticity, and can be polarized differently under different conditions, thereby exhibiting different functionalities [23,24]. The tuberculous granuloma is the main pathological feature of tuberculosis and the most direct site of interaction of *M*. *tuberculosis* with immune cells. Macrophages are the major cell population in the tuberculous granuloma; therefore, a better understanding of macrophage polarization during *M*. *tuberculosis* infection provide insights into the mechanism by which *M*. *tuberculosis* circumvents the host immune system.

Recently, Mattila et al. [15] found that substantial proinflammatory and anti-inflammatory macrophages coexist in the granulomatous lesions in the lung of *M*. *tuberculosis*-infected macaques and tuberculosis patients, suggesting that *M*. *tuberculosis* has the potential to modulate the macrophage polarization. However, this study mainly focused on the observation of phenotypic markers of the macrophages in tuberculous granulomas at a fixed time-point. The variation in functional markers and polarization status of granulomatous macrophages at different stages of infection, and the impact on the formation and development of tuberculous granulomas are still unclear.

In this study, an *in vitro* tuberculous granuloma model was generated based on the reported protocol [17–19], and the phenotypic and functional markers associated with macrophage polarization were determined at different stages of granuloma formation. The results showed that, in the early stages of tuberculous granuloma formation, the polarization status of macrophages was mainly M1 type. The expression levels of M1-associated markers gradually decreased with time, while the M2-associated markers gradually increased. During the late stage of tuberculous granuloma formation in this *in vitro* model, the polarization status of macrophage was mainly M2 type. Thus, this study, for the first time, delineates the sequence of macrophage polarization during tuberculous granuloma formation. Given that modulation of macrophage polarization is one of the main mechanisms by which *M*. *tuberculosis* circumvents the host immune response, leading to immunopathology [25], this study may provide a theoretical foundation for the development of novel therapies of tuberculosis based on reprogramming macrophage polarization.

Recent studies have demonstrated that the molecular markers associated with macrophage polarization are affected by both activators and genetic background [26]. Therefore, before evaluation of macrophage polarization during the course of *M*. *tuberculosis* infection, the classical induction method was applied to determine the phenotypic and functional markers of M1 and M2 macrophages in humans. In brief, IFN-γ+LPS and IL-4 were used to induce transformation of MDM to M1 and M2 types, respectively, and a series of molecular markers were measured. CD86, CXCL11, and TNF-α were selected as M1 molecular markers, while CD206, CCL17, and IL-10 were used as M2 markers in the subsequent studies. However, CD86 expression was persistently lower in the infected macrophages, which was inconsistent with the polarization states reflected by other molecular markers. CD86 is a positive costimulatory molecule and plays an important role in the activation of adaptive immune responses. Thus, it can be speculated that low CD86 expression by *M*. *tuberculosis*-infected cells may be one of the mechanisms by which *M*. *tuberculosis* circumvents the adaptive immune responses of the host. Therefore, we believe that CD86 is not a suitable marker of polarization during the course of *M*. *tuberculosis* infection. In the subsequent human tissue studies, we used iNOS, which is reported to be a M1 phenotypic marker in a several studies [27,28] to ascribe the M1 phenotype.

Our findings in the lung tissues obtained from pulmonary tuberculosis patients showed that iNOS was expressed at low levels, while CD206 was expressed at high levels by macrophages distributed in both the necrotic and non-necrotic granulomas. These findings are in accordance with those reported by Mattila et al. [15], demonstrating M2 dominant activation of macrophages in granulomatous tissues from patients with active tuberculosis. Furthermore, we found that both iNOS and CD206 were expressed at high levels in non-granulomatous lung tissues from pulmonary tuberculosis patients, demonstrating the coexistence of M1 and M2 macrophages in the lung tissues of tuberculosis patients.

Next, we investigated the impact of macrophage polarization on tuberculous granuloma formation as well as the bactericidal effect of macrophages at different polarization states in the *in vitro* granuloma model. The results showed that velocity of granuloma structure formation velocity was similar for macrophages in different polarization states. However, M1 macrophages significantly promoted the formation of tuberculous granuloma, demonstrated by the increase in the number of granuloma-like structures and the increase in macrophage volume. In contrast, IL-4 pretreatment significantly inhibited the formation of tuberculous granulomas. Our study also demonstrated that compared with M0 macrophages, M1 macrophages significantly lowered the *M*. *tuberculosis* load, while M2 macrophages had the opposite effect.

To our knowledge, no previous studies have been reported to investigate whether polarized macrophages influence the formation and development of tuberculous granulomas. Our study, for the first time, reveals that M1 macrophages induced by IFN-γ+LPS promote granuloma formation, while M2 macrophages induced by IL-4 have an inhibitory effect. Our findings further demonstrate that macrophage polarization status plays a critical role in regulating the formation and development of tuberculous granulomas, although the mechanism of this regulation requires further investigation. A recent study by Cyktor et al. revealed that IL-10 inhibited the formation of granulomas induced by *M*. *tuberculosis* in a mouse model [29]. Based on our observation that IL-10 was greatly elevated in the M2 macrophage model induced by IL-4, it can be speculated that the inhibitory role of M2 macrophages in preventing the formation and development of granulomas may be partially mediated by IL-10.

In summary, our findings demonstrate the coexistence of M1 and M2 polarized macrophages in the tuberculous granuloma and indicate that M1 macrophages promote tuberculous granuloma formation, while M2 macrophages inhibit this process.
